# Effects of konjac gel with vegetable powders as fat replacers in frankfurter-type sausage

**DOI:** 10.5713/ajas.18.0781

**Published:** 2019-01-04

**Authors:** Dong Hyun Kim, Dong Min Shin, Han Geuk Seo, Sung Gu Han

**Affiliations:** 1 Department of Food Science and Biotechnology of Animal Resources, Konkuk University, Seoul 05029, Korea

**Keywords:** Low-fat Frankfurter, Fat Replacer, Konjac Gel, Cactus Pear, Aloe Vera, Wheat Sprout

## Abstract

**Objective:**

The purpose of this study was to investigate whether addition of konjac gel with three different vegetable powders can increase quality of low-fat frankfurter-type sausage.

**Methods:**

Low-fat frankfurter-type sausages were manufactured with formulations containing konjac gel and three vegetable powders (aloe vera, cactus pear, or wheat sprout) as pork fat replacers. The formulations of frankfurters were as follows: NF (normal-fat; 20% pork fat), LF (low-fat; 10% pork fat), KG (low-fat; 10% pork fat+10% konjac gel), and konjac gel with three vegetable powders (KV), such as KV-AV (10% pork fat+10% konjac gel with aloe vera), KV-CP (10% pork fat+10% konjac gel with cactus pear), and KV-WS (10% pork fat+10% konjac gel with wheat sprout). Proximate analysis, pH value, color evaluation, cooking loss, water-holding capacity, emulsion stability, apparent viscosity, texture profile analysis, and sensory evaluation were determined.

**Results:**

The konjac gel containing groups showed lower fat content (p<0.05) and higher moisture content than NF group (p<0.05). The pH value of frankfurters was decreased in three KV groups (p<0.05). The three KV groups had increased dark color (p<0.05) compared with KG, and KV-CP had the highest redness (p<0.05). The water-holding capacity and emulsion stability were higher in the three KV groups than KG and LF (p<0.05). Cooking loss was generally decreased in the three KV groups, compared with KG (p<0.05). The apparent viscosity of KV groups was similar with NF group and overall texture properties were improved in KV-CP. In the sensory evaluation, the highest overall acceptability was found in KV-CP groups (p<0.05).

**Conclusion:**

The four fat replacers improved physicochemical properties of low-fat frankfurters. Particularly, konjac gel with cactus pear powder seems more acceptable as a pork fat replacer.

## INTRODUCTION

Fat plays important roles in meat products by reducing cooking loss, forming meat emulsions, and improving water holding capacity (WHC), hardness, juiciness and mouth feel [1]. Fat is the principal source of vitamin A and D, and essential fatty acids such as omega-3 and -6 [2]. However, excessive fat intake is also associated with health problems such as coronary heart disease, cardiovascular diseases, hypertension and obesity [1]. Thus, there is a need for developing low-fat foods to lower health problems and concerns [3]. In this regard, substitutes for animal fat have been studied and some examples were isolated soy protein, vegetable oil, dietary fiber, konjac and carrageenan [1,4,5].

Konjac (*Amorphophallus konjac*), a water-soluble polysaccharide, is one of the suggested animal fat replacers. The glucomannan from konjac has bioactive effects for obese people by delaying gastric emptying and as a hypocholesterolmic agent by interfering the transportation of cholesterol in body [6]. Konjac gel showed synergistic and improved physiological properties by combining with other additives, such as polysaccharide and protein [7]. Konjac gel combined with other food additives has been used to improve texture and structure stability of meat products [8]. However, several studies also mentioned that meat products with konjac gel cause higher cooking loss, lower WHC, and lower emulsion stability [9–11].

Among a wide variety of natural ingredients, some vegetable powders have been employed in manufacturing meat products. Addition of aloe vera (*Aloe barbadensis Miller*) into meat products decreased the cooking loss, and increased WHC and emulsion stability [12–14]. In meat products, cactus pear (*Opuntia ficus-indica* var. *saboten*) improved WHC and decreased expressible moisture [15,16]. Additionally, wheat sprout improved both the quality and physicochemical properties of meat products [5].

Therefore, the combined use of vegetable powders (i.e., aloe vera, cactus pear or wheat sprout) and konjac gel might be a good strategy to be tested in the development of low-fat meat products. Thus, the aims of this study were: i) to manufacture frankfurter sausage added with konjac gels with each vegetable powder (refined aloe vera, cactus pear and wheat sprout), ii) to investigate the effects of konjac gels with vegetable powders as a fat replacer for low-fat frankfurters by measuring proximate component, pH, color, texture, WHC, cooking loss, emulsion stability, viscosity and sensory evaluation.

## MATERIALS AND METHODS

### Preparation of raw materials for manufacturing sausages

Fresh pork ham (*Musculus Biceps femoris*, M. *Semitendinosus*, and M. *Semimembranosus*, moisture 72.47%, fat 5.02%, protein 15.77%) and pork back fat (moisture 10.35%, fat 85.83%) were purchased from a local processor at post-mortem 48 h. All subcutaneous and intramuscular fat, and visible connective tissue were removed from the fresh pork muscle. Four types of konjac flour gel were prepared in accordance with modified method of Jimenez-Colmenero et al [4]. Briefly, for four 500 g of konjac gel preparation, konjac flour (25 g) and k-carrageenan (5 g) were homogenized (Model AM-7, Nihonseiki Kaisha Ltd., Tokyo, Japan) in 324 mL of water at 3,000 rpm for 6 min. The 15 g of water or each vegetable powder (aloe vera, cactus pear, or wheat sprout; all obtained from a local market) and 81 mL of water were added to the konjac gel and then homogenized at 3,000 rpm for 3 min. The mixed konjac gels with each vegetable powder were cooled to 10°C, then 50 mL of cold water was added with gentle stirring at room temperature. They were maintained at below 10°C during gel formation for 24 h.

### Formulation and processing of sausages

Both lean materials and pork back fat were minced through an 8 mm plate. The ground tissue was then placed in polyethylene bags, vacuum packaged using a vacuum packaging system (FJ-500XL, Fujee Tech., Seoul, Korea) and stored at 0°C until manufacturing products. Compositions of six different meat batters (300 g batches of each meat batters) are shown in Table 1. The normal-fat formulation (NF) was produced with 20% of added pork backfat and 20% of water. Low-fat formulation (LF) was made with 10% of added pork back fat and 30% of water. Konjac gel formulation (KG) was prepared with 10% of added pork back fat and 10% of konjac gel. Three konjac gel with vegetable powder (KV) groups (i.e., back fat replacers) were as follows: KV-AV (10% pork fat+10% konjac gel with aloe vera), KV-CP (10% pork fat+10% konjac gel with cactus pear), and KV-WS (10% pork fat+10% konjac gel with wheat sprout). Pork meat was homogenized and ground for 1 min in a silent cutter (Cutter Nr-963009, Scharfen, Postfach, Germany). Ground meat (60%), NaCl (1.3%), and sodium tripolyphosphate (0.3%) were mixed for 1 min. Pork back fat or back fat replacer was added to the meat for 3 min and the batters were mixed for 6 min with 0.5% sugar. The temperature of the emulsion was monitored by temperature probe (KM330, Kane-May, Harlow, UK) below 10°C during batter preparation. After emulsification, meat batters were filled into collagen casings (#240, NIPPI Inc., Tokyo, Japan; approximate 25 mm diameter) by using a stuffer (IS-8, Sirman, Marsango, Italy). The frankfurters were cooked at 75°C for 30 min in a water bath (Model 10–101, Dae Han Co., Seoul, Korea). The cooked samples were cooled using cold water (15°C), and then analyzed for physicochemical and textural properties. All treatment groups, approximately 5 kg each, were replicated with three times at three different days.

### Proximate composition

Compositional properties of vegetable powders and frankfurter sausages were performed using AOAC [17]. The results are shown in Table 2 and 3. Moisture content was measured by weight loss after 12 h of drying at 105°C in a drying oven (SW-90D, Sang Woo Scientific Co., Bucheon, Korea). Fat content was determined by the Soxhlet method with a solvent extraction system (Soxtec Avanti 2050 Auto System, Foss Tecator AB, Höganas, Sweden). Protein content was determined by Kjeldahl method with an automatic Kjeldahl nitrogen analyzer (Kjeltec 2300 Analyzer Unit, Foss Tecator AB, Sweden). Ash was determined by incinerating samples at 550°C in a muffle furnace. The available carbohydrate of vegetable powder was estimated by subtracting the per cent total of the moisture, fat, protein and ash contents from 100.

### pH

The pH values of samples were measured in a homogenate (Ultra-Turrax T25, Janke and Kunkel Staufen, Germany) prepared with 5 g of sample with 20 mL distilled water and the pH of those homogenates was determined with a pH meter (Model 340, Mettler-Toledo GmbH, Schwerzenbach, Switzerland). All determinations were performed in triplicate.

### Color evaluations

The color of frankfurter sausages and vegetable powders were determined using a colorimeter (Minolta Chroma meter CR-210, Japan; illuminate C, calibrated with white plate, L* = +97.83, a* = −0.43, b* = +1.98). L* (lightness), a* (redness), and b* (yellowness) values were measured on the surface of raw batters and cooked frankfurter sausages. Five measurements were obtained in each of six replicated.

### Cooking loss

Each raw batter was stuffed into a collagen casing and cooked at 75°C for 30 min. Cooking loss was determined by calculating the differences in weight before and after cooking as follows:

Cooking loss (g/100 g)={[(weight of raw batter (g)-weight of cooked frankfurter sausage (g)]/weight of raw batter (g)} ×100

### Emulsion stability

The emulsion stability was carried out using the method of Choi et al [1]. At the middle of a 15 mesh sieve, pre-weighed graduated glass tubes were filled with raw batter. The glass tubes were enveloped and heated in a 75°C water bath to for 30 min. After cooling to approximately 4°C to facilitate fat and water layer separation, the total expressible fluid and fat separated in the bottom of graduated glass tube were measured calculated as follows:

$$\begin{array}{l} \text{Water released }(\%)=[\text{the water layer }(\text{mL})/\text{weight of raw sausage }(\text{g})]\times 100\\ \text{Fat released }(\%)=[\text{the fat layer }(\text{mL})/\text{weight of raw sausage }(\text{g})]\times 100 \end{array}$$

### Water released (%) = [the water layer (mL)/weight of raw sausage (g)]×100

Fat released (%) = [the fat layer (mL)/weight of raw sausage (g)]×100

### Water holding capacity

The WHC of raw batter was determined with modificated method of Yang et al [18]. Approximately 10 g of the sausage was placed in the centrifuge tube and centrifuged for 15 min at 6,000 g at 4°C. The samples were heated in a water bath at 90°C for 15 min and then removed, cooled to room temperature. After cooling, the samples were centrifuged for 15 min at 6,000 *g* at 4°C, removed, and weighed. The WHC was calculated as follows:

WHC (%)=[1-(BW-AW)/WC]×100

BW = weight of sample before heating and centrifugation (g)
AW = sample after heating and centrifugation (g)
WC = water content in the sample (g)

### Apparent viscosity

The viscosity was measured with a rotational viscometer (HAKKE Viscotester 500, Thermo Elctron Corporation, Karlsruhe, Germany) at 10 rpm in triplicated. The standard cylinder sensor (SV-2) was positioned in a 50 mL metal cup filled with sausage and allowed to rotate under a constant share rate at s^−1^ for 30 s before each reading was taken. Apparent viscosity values in centipoises were determined. The temperature of each sample at the time (18°C±1°C) of viscosity testing was also recorded [19].

### Texture profile analysis

Texture properties of frankfurter sausages were performed at room temperature with a texture analyzer (TA-XT2*i*, Stable Micro Systems, Surry, UK). The central core of samples was cut with sized 2.5×2.0 cm (diameter×length). After samples were cooled to room temperature (20°C, 3 h), texture profile analysis (TPA) was measured. The conditions of texture analysis were as follows: pre-test speed 2.0 mm/s, post-test speed 4.0 mm/s, maximum load 2 kg, head speed 2.0 mm/s, distance 8.0 mm, force 5 g [1]. Values for hardness (*N*), springiness, cohesiveness, gumminess (*N*), and chewiness (*N*), were determined as described [20].

### Sensory evaluation

The sensory evaluations were performed in triplicate on each sample. Each frankfurter sausage was evaluated in terms of color, flavor, odor, texture, juiciness, saltiness, and overall acceptability. The selected sensory panel consisted of 10 researchers from the Department of Food Sciences and Biotechnology of Animal Resources at Konkuk University. Frankfurters were cooked to a center temperature of 75°C, cooled to 21°C, sliced into pieces of approximately 1 cm thickness and identified with a random code. Sensory evaluations were placed on the panelists under fluorescent lighting. Panelists were instructed to cleanse their palates between samples using water for gargling. The color, flavor, odor, texture, juiciness, saltiness, and overall acceptability were evaluated using a 10-point descriptive scale (1 = extremely undesirable, 10 = extremely desirable) [21].

### Statistical analysis

All tests were done three times for each experimental condition, and mean values were reported. An analysis of variance was performed on all the variables measured using SPSS Ver. 20.0 (SPSS Inc., Chicago, IL, USA). The Duncan’s multiple range tests was used to determine the differences among treatments (p<0.05).

## RESULTS AND DISCUSSION

### Physicochemical properties of vegetable powders

The physicochemical properties of vegetable powder are shown in Table 2. The highest moisture content was observed in cactus pear powder, compared the other vegetable powders (p< 0.05). The protein content was highest in wheat sprout powder (p<0.05). Cactus pear powder showed the highest fat content (p<0.05). The ash and carbohydrate contents of aloe vera was highest among the three vegetable powders (p<0.05).

The lightness (L*-value), redness (a*-value), yellowness (b*-value) and pH values of vegetable powders are also demonstrated in Table 2. The lightness and yellowness value of cactus pear powder were lowest among vegetable powders (p<0.05), whereas the redness was highest compared to other powders (p<0.05). The pH value of wheat sprout powder was higher than the other vegetable powders (p<0.05).

### Proximate composition

The proximate composition was affected by formulation such as fat level and konjac gels added with vegetable powders (Table 3). The groups containing konjac gels had more moisture contents than the NF (p<0.05). The result agrees with those of Osburn and Keeton [11], in which the moisture content increased with replacement of fat with the addition of different levels of konjac gel to low-fat sausage. Similarly, Jimenez-Colmenero et al [7] observed that the moisture content of dry fermented sausage was increased by addition of konjac gel and reduction of fat. The highest protein content was observed in the NF compared to the other treatments (p<0.05). Utilization of konjac gel as a fat substitute decreased protein content in low-fat sausage by Osburn and Keeton [11]. The different of protein content between NF and low-fat treatments could be due to the protein contribution from pork fat. The fat content of konjac gel added groups and of LF group were lower than that of the NF sample and there were no significant differences among the various konjac gel treatments and LF group (p<0.05). According to Delgado-Pando et al [9], the addition of oil combination and konjac gel to pates showed the reduction of fat content because of formulation differences.

### pH and color evaluation

Addition of konjac gels and vegetable powders significantly affected the pH, lightness (L*-value), redness (a*-value), and yellowness (b*-value) values of uncooked and cooked frankfurter sausages (Table 4). The pH value of sausages and batters was decreased by replacing 50% pork fat with konjac gel (p< 0.05). KV-AV and KV-CP groups exhibited lower pH value than KV-WS groups (p<0.05). This is because addition of aloe vera powder (pH 4.63 in 0.025% solution) and cactus pear powder (pH 3.99 in 0.025% solution) lowered the pH value of frankfurter. Soltanizadeh and Ghiasi-Esfahani [14] also found that addition of aloe vera resulted in lower pH in beef burgers. Similarly, Jin et al [16] reported that the pH value of sausages could be decreased using cactus pear powder.

The lightness of cooked and uncooked frankfurters was highest in the KG group and KV-CP group showed relatively lower lightness among groups (p<0.05). The decreased lightness was observed in the meat products as reduction of fat or addition of colored vegetable powders [5,9, 14,15]. The redness value of uncooked and cooked sausage was highest in sausages with cactus pear powder (p<0.05), whereas the yellowness was higher in groups of KV-AV and KV-WS (p<0.05). Lee et al [22] added ethanol extract of cactus pear and lentil bean to breakfast sausages and the results showed decreasing lightness while increasing redness by adding increasing amount of cactus pear. Addition of cactus pear contributed to the highest a*-value, and this is maybe due to redness of betanin in cactus pear. Our data showed higher yellowness value in KV-WS group and these data agreed with a previous report where wheat sprout caused increased yellowness and decreased lightness and redness in reduced-fat chicken patties [5]. The color of wheat sprout and aloe vera seems to influence on the b*-value of KV-AV and KV-WS.

### Cooking loss and emulsion stability

Table 5 shows the cooking loss of frankfurters formulated with konjac gels and vegetable powders. Cooked frankfurters formulated with konjac gel (i.e., KG and KV groups) had significantly lower cooking loss than LF groups (p<0.05). It seems the addition of konjac gel to low-fat sausage (KG) helps preventing cooking loss of low-fat sausage (LF) but still had higher cooking loss than normal fat sausage (NF). NF group showed the lowest cooking loss among all the groups (p<0.05) and also both KV-AV and KV-CP also showed lower cooking loss as NF group (p<0.05). Jimenez-Colmenero et al [4] reported different results where frankfurters with konjac gel increased cooking loss compared to normal-fat controls. Also, Osburn and Keeton [11] have shown that incorporation of 0% to 20% konjac gel as a fat replacer into low-fat cured sausage increased cooking loss. In fact, the high moisture content in LF and groups with konjac gel (Table 3) may resulted in increased cooking loss, compared with the NF group (p<0.05). However, the addition of konjac gel with cactus pear and aloe vera powder displayed a minimal cooking loss which is statistically non-significant compared with NF group (p<0.05). According to Diaz-Vela et al [15], the addition of cactus pear peel flour enhanced sausage yield in cooked meat products.

The effects of konjac gels with vegetable powders on the emulsion stability in frankfurters are also shown in Table 5. The total released fluid was lower in frankfurters formulated with konjac gels than LF (p<0.05). Addition of aloe vera powder to konjac gel (KV-AV) showed the highest emulsion stability (p<0.05). The konjac gel groups (KG, KV-AV, and KV-CP) showed the lowest fat separation (p<0.05). In pates containing konjac gel and oil combination (olive, linseed and fish oils), Delgado-Pando et al [9] reported high expressible fluid fat and water in low-fat combined with konjac gel and oils, as comparing normal-fat samples. The frankfurters with konjac gel which replacing 50% of pork backfat did not affect the emulsion stability of the reduced-fat product but konjac gel which replacing 92% of pork backfat produced increased release of water and fat [23]. Our data showed improved emulsion stability in KV groups because 50% of back fat was replaced. In addition, vegetable powder may contribute to the stabilization of the emulsion. Our data is supported by a previous report where the meat emulsion with aloe gel in the low-fat formulations improved the emulsion stability by lowering the released water and fat [24]. Bhat et al [12] observed an increased emulsion stability of chicken nuggets by incorporation of 5, 10, and 15 percent aloe vera pulp. In addition, water and fat retention capacity in meat products were improved because of high contents of total dietary fiber in cactus pear [15]. Based on these past and our data, the high emulsion stability and low cooking loss in frankfurter were contributed by konjac gel and vegetable powders.

### Water holding capacity

**Figure 1:**
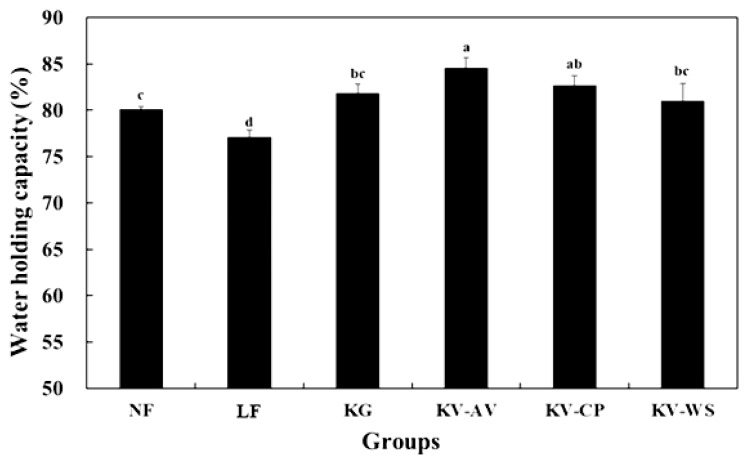
Water holding capacity of frankfurter sausages. Groups of frankfurter sausage: NF, normal-fat frankfurter sausage formulated with 20% ice and 20% fat; LF, low-fat frankfurter sausage formulated with 30% ice and 10% back fat; KG, frankfurter sausage formulated with 10% konjac gel and 10% back fat; KV-AV, frankfurter sausage formulated with 10% konjac gel with aloe vera vegetable powder and 10% back fat; KV-CP, frankfurter sausage formulated with 10% konjac gel with cactus pear vegetable powder and 10% back fat; KV-WS, frankfurter sausage formulated with 10% konjac gel with wheat sprout vegetable powder and 10% back fat. ^a–d^ Means in the treatments with different letters are significantly different (p<0.05).

The WHC was affected by the formulation of frankfurters (Figure 1). The WHC was lowest in the LF which showed the highest cooking loss and released water and fat in Table 5 (p<0.05). The frankfurters with konjac gels had greater WHC, compared to those without konjac gels (p<0.05). In a previous report about the cooked beef sausage, fat reduction to 5% resulted in lower emulsion stability and WHC and higher cooking loss than 20% fat sausage [25]. However, this past study showed the addition of soy protein, whey powder and wheat gluten into the beef sausage contributed to decrease of cooking loss, and improved water holing capacity and emulsion stability. Yang et al [18] suggested that addition of hydrated oatmeal into low-fat sausages improve WHC by decreasing cooking loss. In our study, among groups with konjac gels, KV-AV showed the highest WHC (p<0.05). Scala et al [13] found WHC of aloe vera is around 77.72% (77.72 g retained water/100 g water). Soltanizadeh and Ghiasi-Esfahani [14] reported that low meat burgers containing aloe vera showed higher WHC than normal meat burger samples. KV-CP group showed a decent WHC as KV-AV. A previous report about WHC of cactus pear in meat products may confirm our data [16]. In summary, the combination of konjac gel and vegetable powders, particularly aloe vera and cactus pear, could improve WHC and emulsion stability and thus decreasing cooking loss, compared to the other treatments.

### Apparent viscosity

**Figure 2:**
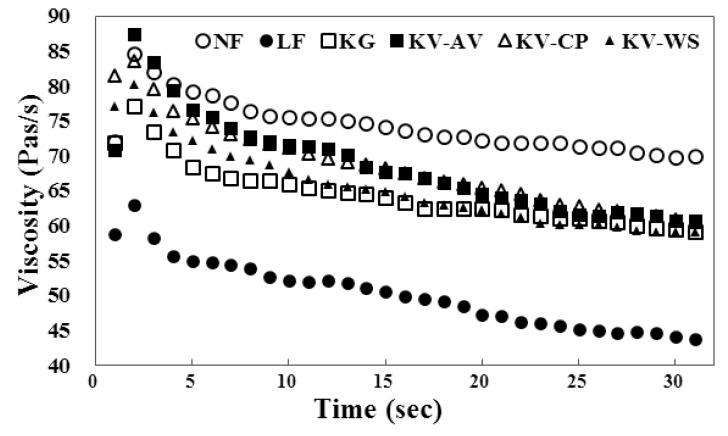
Apparent viscosity of frankfurter sausages. Groups of frankfurter sausage: NF, normal-fat frankfurter sausage formulated with 20% ice and 20% fat; LF, low-fat frankfurter sausage formulated with 30% ice and 10% back fat; KG, frankfurter sausage formulated with 10% konjac gel and 10% back fat; KV-AV, frankfurter sausage formulated with 10% konjac gel with aloe vera vegetable powder and 10% back fat; KV-CP, frankfurter sausage formulated with 10% konjac gel with cactus pear vegetable powder and 10% back fat; KV-WS, frankfurter sausage formulated with 10% konjac gel with wheat sprout vegetable powder and 10% back fat.

The apparent viscosity of frankfurters was significantly decreased in LF group but the incorporation of konjac gel with vegetable powders restored the lowered viscosity (Figure 2). Results showed KV-AV, NF, and KV-CP had relatively higher viscosity values, compared to the other groups. KG showed the lowest viscosity among konjac gel containing groups. LF group displayed the lowest viscosity. Choi et al [1] showed that high fat content can provide the meat batters for higher emulsion and viscosity than low-fat samples. Also, addition of rice bran fiber and vegetable oil could improve viscosity in low-fat meat products. An increase in meat gel viscosity is associated with increasing dietary fiber of rice bran because this fiber provided higher elasticity in emulsion-type meat products [1]. Lee et al [5] showed that wheat sprout powder added at breakfast sausage enhanced the apparent viscosity value as compared to the samples without wheat sprout powder. These past results and our data indicate that incorporation of konjac gel and fiber-containing vegetable powders into frankfurters can improve gel strength and viscosity in meat products.

### Texture profile analysis

The addition of various konjac gels affected the apparent texture profile of frankfurters (Table 6). KV-AV had the lowest hardness, springiness and gumminess compared with other groups (p<0.05). Besides the KV-AV group, the texture parameter values were not markedly different among all groups. Our results indicate that konjac gels with vegetable powders, particularly aloe vera powder, can affect not only the stability of water and protein matrix but also the textures of low-fat frankfurter.

### Sensory evaluation

As expected, the partial replacement of pork backfat by konjac gel with vegetable powders affected the sensory evaluation of the frankfurters (Table 7). The KV-CP group was significantly higher in color score in sensory evaluation, compared to the other groups (p<0.05). Jin et al [16] reported that sausage with 10% cactus pear powder had higher redness than sausage with 0.014% sodium nitrite and there was no significant different in color score from sensory evaluation. Flavor score was the highest in the KV-CP group (p<0.05). The odor obtained for the KV-AV were significantly lower than for the other groups (p<0.05), but there was no significant difference among the other groups (p<0.05). Chua et al [26] reported that crude konjac flour has a fish-like smell and an acrid taste. Chin et al [27] showed that the use of konjac on low-fat sausage could decrease flavor score of sensory attributes. Like this previous report, incorporation of konjac gel (KG) led to insignificant decrease of flavor compared to LF. The flavor score was higher in KV-CP and this result is similar in a past report showing high flavor score of cooked sausage by cactus pear due to its delicate flavor [16]. However, Kumar et al [24] showed that the low-fat meat emulsion produced low flavor attribute with the addition of 5% aloe gel. Similar findings on low flavor acceptability scores have been reported by Soltanizadeh and Ghiasi-Esfahani [14] in low meat beef burger with aloe vera and this is probably due to their aloin with a bitter taste. The tenderness (texture), juiciness and saltiness scores were nonsignificant among all groups. The overall acceptability of frankfurter was the greatest in KV-CP groups (p<0.05). This result may be influenced by color and flavor of the cactus pear. Thus, cactus pear konjac gel could be used for the fat replacer of frankfurters with positive effect on the color, flavor, odor, and overall acceptability.

## IMPLICATION

Addition of vegetable konjac gels in the formulation improved overall quality of the WHC, cooking loss and emulsion stability. Apparent viscosity and TPA were significantly improved in low-fat frankfurters with konjac gels containing aloe vera, cactus pear or wheat sprout powder, compared to the low-fat frankfurter. The replacement of 50% pork fat by konjac gel with cactus pear powder showed positive impacts on cooking loss, emulsion stability, texture properties, WHC, viscosity and overall sensory attributes. Based on these results, addition of konjac gel with cactus pear seems to improve physicochemical properties and also acceptable in sensory properties in low-fat sausage.

## Figures and Tables

**Table 1:** Formulations of frankfurter sausages

Ingredients (%)	Treatments1)					

	NF	LF	KG	KV-AV	KV-CP	KV-WS
Pork meat	60	60	60	60	60	60
Ice	20	30	20	20	20	20
Back fat	20	10	10	10	10	10
Konjac gel or konjac gel with vegetable powder	-	-	10	10	10	10
Total	100	100	100	100	100	100
Sodium chloride (NaCl)	1.3	1.3	1.3	1.3	1.3	1.3
Tripolyphosphate	0.3	0.3	0.3	0.3	0.3	0.3
Sugar	0.5	0.5	0.5	0.5	0.5	0.5

1) NF, normal-fat frankfurter sausage formulated with 20% ice and 20% fat; LF, low-fat frankfurter sausage formulated with 30% ice and 10% back fat; KG, frankfurter sausage formulated with 10% konjac gel and 10% back fat; KV-AV, frankfurter sausage formulated with 10% konjac gel with aloe vera vegetable powder and 10% back fat; KV-CP, frankfurter sausage formulated with 10% konjac gel with cactus pear vegetable powder and 10% back fat; KV-WS, frankfurter sausage formulated with 10% konjac gel with wheat sprout vegetable powder and 10% back fat.

**Table 2:** The proximate composition, color and pH of vegetable powders

Properties	Vegetable powders		

	Aloe vera	Cactus pear	Wheat sprout
Proximate composition			
Moisture (%)	6.58±0.24^b^	8.97±0.39^a^	4.29±0.29^c^
Protein (%)	5.58±0.55^c^	8.16±0.52^b^	15.01±0.12^a^
Fat (%)	2.37±0.10^c^	6.90±0.09^a^	3.44±0.22^b^
Ash (%)	8.07±0.02^a^	7.92±0.02^b^	5.79±0.01^c^
Carbohydrates (%)	77.43±0.61^a^	68.06±0.17^c^	71.47±0.69^b^
Color			
L*-value	63.26±1.50^c^	31.52±1.54^b^	68.36±1.66^a^
a*-value	−1.82±0.17^a^	34.29±0.24^b^	−6.40±0.12^c^
b*-value	26.73±0.29^c^	8.27±0.14^a^	25.52±0.60^b^
pH at 25°C (0.025% sol.)	4.63±0.01^b^	3.99±0.01^c^	5.49±0.01^a^

All values are mean±standard deviation of three replicates.

a–c Means within a row with different letters are significantly different (p<0.05).

**Table 3:** The proximate composition of frankfurter sausages

Items	Treatments1)					

	NF	LF	KG	KV-AV	KV-CP	KV-WS
Moisture (%)	61.90±0.63^c^	69.30±0.46^ab^	70.21±0.89^a^	69.73±0.59^ab^	68.81±0.21^b^	69.2±1.00^b^
Protein (%)	14.15±0.17^a^	13.93±0.13^ab^	13.74±0.24^bc^	13.57±0.18^c^	13.94±0.17^ab^	13.82±0.21^bc^
Fat (%)	22.66±0.79^a^	14.25±0.48^b^	14.25±0.64^b^	14.43±0.40^b^	14.55±0.34^b^	14.63±0.17^b^
Ash (%)	2.16±0.01	2.21±0.06	2.20±0.06	2.26±0.09	2.23±0.03	2.22±0.08

All values are mean±standard deviation of three replicates.

1) NF, normal-fat frankfurter sausage formulated with 20% ice and 20% fat; LF, low-fat frankfurter sausage formulated with 30% ice and 10% back fat; KG, frankfurter sausage formulated with 10% konjac gel and 10% back fat; KV-AV, frankfurter sausage formulated with 10% konjac gel with aloe vera vegetable powder and 10% back fat; KV-CP, frankfurter sausage formulated with 10% konjac gel with cactus pear vegetable powder and 10% back fat; KV-WS, frankfurter sausage formulated with 10% konjac gel with wheat sprout vegetable powder and 10% back fat.

a–c Means within a row with different letters are significantly different (p<0.05).

**Table 4:** The pH and color parameters of frankfurter sausages

Items	Parameters	NF1)	LF1)	KG1)	KV-AV1)	KV-CP1)	KV-WS1)
Uncooked	pH	6.42±0.01^a^	6.41±0.01^a^	6.33±0.01^b^	6.23±0.01^d^	6.11±0.01^e^	6.25±0.01^c^
	L*-value	67.46±1.41^b^	62.52±0.75^d^	70.60±0.86^a^	65.19±1.92^c^	63.69±0.70^cd^	68.79±1.50^b^
	a*-value	12.92±0.32^b^	12.98±0.70^b^	13.41±0.79^b^	10.20±0.78^c^	23.90±0.69^a^	9.73±0.58^c^
	b*-value	14.04±0.75^c^	13.17±0.76^c^	15.68±0.46^b^	17.65±1.02^a^	13.93±0.75^c^	17.43±0.54^a^
Cooked	pH	6.43±0.01^b^	6.47±0.01^a^	6.40±0.01^c^	6.36±0.01^e^	6.35±0.01^e^	6.38±0.01^d^
	L*-value	71.16±1.43^bc^	72.29±1.70^ab^	73.41±0.90^a^	71.58±0.76^b^	69.83±1.14^c^	71.93±0.60^b^
	a*-value	4.60±0.66^b^	4.15±0.28^bc^	4.10±0.39^bc^	3.48±0.58^c^	9.06±0.92^a^	2.03±0.11^d^
	b*-value	9.91±0.79^c^	9.76±0.38^c^	10.83±0.55^b^	12.24±0.75^a^	10.68±0.92^b^	12.77±0.17^a^

All values are mean±standard deviation of three replicates.

1) NF, normal-fat frankfurter sausage formulated with 20% ice and 20% fat; LF, low-fat frankfurter sausage formulated with 30% ice and 10% back fat; KG, frankfurter sausage formulated with 10% konjac gel and 10% back fat; KV-AV, frankfurter sausage formulated with 10% konjac gel with aloe vera vegetable powder and 10% back fat; KV-CP, frankfurter sausage formulated with 10% konjac gel with cactus pear vegetable powder and 10% back fat; KV-WS, frankfurter sausage formulated with 10% konjac gel with wheat sprout vegetable powder and 10% back fat.

a–e Means within a row with different letters are significantly different (p<0.05).

**Table 5:** Cooking loss and emulsion stability of frankfurter sausages

Parameters		NF1)	LF1)	KG1)	KV-AV1)	KV-CP1)	KV-WS1)
Cooking loss (%)		4.55±0.36^d^	7.19±0.91^a^	5.43±0.44^bc^	5.17±0.14^cd^	5.05±0.54^cd^	5.91±0.24^b^
Emulsion stability of meat batter	Total released (%)	6.99±0.83^b^	13.50±1.33^a^	5.89±0.68^bc^	5.49±0.01^c^	5.89±0.23^bc^	6.29±0.60^bc^
	Water released (%)	6.19±0.83^b^	12.60±1.30^a^	5.49±0.68^b^	5.09±0.38^b^	5.49±0.38^b^	5.69±0.68^b^
	Fat released (%)	0.80±0.01^a^	0.90±0.20^a^	0.40±0.01^b^	0.40±0.07^b^	0.40±0.38^b^	0.60±0.23^b^

All values are mean±standard deviation of three replicates.

1) Treatments: NF, normal-fat frankfurter sausage formulated with 20% ice and 20% fat; LF, low-fat frankfurter sausage formulated with 30% ice and 10% back fat; KG, frankfurter sausage formulated with 10% konjac gel and 10% back fat; KV-AV, frankfurter sausage formulated with 10% konjac gel with aloe vera vegetable powder and 10% back fat; KV-CP, frankfurter sausage formulated with 10% konjac gel with cactus pear vegetable powder and 10% back fat; KV-WS, frankfurter sausage formulated with 10% konjac gel with wheat sprout vegetable powder and 10% back fat.

a–d Means within a row with different letters are significantly different (p<0.05).

**Table 6:** Texture profile analysis of frankfurter sausages

Parameters	NF1)	LF1)	KG1)	KV-AV1)	KV-CP1)	KV-WS1)
Hardness (*N*)	3.57±0.42^a^	3.56±0.31^a^	3.65±0.16^a^	2.69±0.31^b^	3.69±0.59^a^	3.59±0.28^a^
Springiness	1.46±0.30^b^	1.56±0.11^ab^	1.61±0.12^a^	1.02±0.10^c^	1.61±0.21^ab^	1.55±0.08^ab^
Cohesiveness	0.97±0.01^b^	0.96±0.05^b^	1.00±0.01^ab^	0.96±0.03^b^	0.99±0.01^ab^	1.01±0.03^a^
Gumminess (*N*)	1.36±0.28^b^	1.49±0.06^ab^	1.61±0.14^a^	0.44±0.06^c^	1.57±0.19^a^	1.57±0.08^a^
Chewiness (*N*)	0.39±0.06^c^	0.44±0.03^a^	0.44±0.03^a^	0.40±0.01^bc^	0.43±0.02^ab^	0.44±0.02^a^

All values are mean±standard deviation of three replicates.

1) NF, normal-fat frankfurter sausage formulated with 20% ice and 20% fat; LF, low-fat frankfurter sausage formulated with 30% ice and 10% back fat; KG, frankfurter sausage formulated with 10% konjac gel and 10% back fat; KV-AV, frankfurter sausage formulated with 10% konjac gel with aloe vera vegetable powder and 10% back fat; KV-CP, frankfurter sausage formulated with 10% konjac gel with cactus pear vegetable powder and 10% back fat; KV-WS, frankfurter sausage formulated with 10% konjac gel with wheat sprout vegetable powder and 10% back fat.

a–c Means within a row with different letters are significantly different (p<0.05).

**Table 7:** Sensory properties of frankfurter sausages

Parameters	NF1)	LF1)	KG1)	KV-AV1)	KV-CP1)	KV-WS1)
Color	5.82±0.98^b^	4.82±0.87^c^	5.18±0.75^bc^	4.91±0.83^c^	7.55±0.52^a^	4.70±1.06^c^
Flavor	5.45±1.04^ab^	5.73±0.90^ab^	5.55±0.82^ab^	4.36±0.81^c^	6.00±0.77^a^	4.91±0.94^bc^
Odor	6.36±1.03^a^	6.00±1.00^a^	6.09±1.04^a^	4.73±0.79^b^	6.36±1.03^a^	5.64±0.81^a^
Texture	6.09±0.94	5.82±0.87	5.91±0.83	5.09±0.83	5.82±0.87	5.64±0.92
Juiciness	5.91±0.70	6.18±0.98	5.82±0.87	5.55±0.82	6.00±0.89	5.64±0.81
Saltiness	5.20±0.92	5.27±0.79	5.27±0.65	4.80±0.92	5.09±0.70	5.00±0.89
Overall acceptability	6.00±1.05^ab^	6.00±0.94^ab^	6.10±0.99^ab^	4.70±0.82^c^	6.70±0.95^a^	5.30±1.06^bc^

All values are mean±standard deviation of three replicates.

1) NF, normal-fat frankfurter sausage formulated with 20% ice and 20% fat; LF, low-fat frankfurter sausage formulated with 30% ice and 10% back fat; KG, frankfurter sausage formulated with 10% konjac gel and 10% back fat; KV-AV, frankfurter sausage formulated with 10% konjac gel with aloe vera vegetable powder and 10% back fat; KV-CP, frankfurter sausage formulated with 10% konjac gel with cactus pear vegetable powder and 10% back fat; KV-WS, frankfurter sausage formulated with 10% konjac gel with wheat sprout vegetable powder and 10% back fat.

a–c Means within a row with different letters are significantly different (p<0.05).
